# Properties of Poly (Lactic-co-Glycolic Acid) and Progress of Poly (Lactic-co-Glycolic Acid)-Based Biodegradable Materials in Biomedical Research

**DOI:** 10.3390/ph16030454

**Published:** 2023-03-17

**Authors:** Yue Lu, Dongfang Cheng, Baohua Niu, Xiuzhi Wang, Xiaxia Wu, Aiping Wang

**Affiliations:** 1 Collaborative Innovation Center of Advanced Drug Delivery System and Biotech Drugs in Universities of Shandong, Key Laboratory of Molecular Pharmacology and Drug Evaluation, Ministry of Education, School of Pharmacy, Yantai University, Yantai 264005, China; 2 Yantai Key Laboratory of Nanomedicine and Advanced Preparations, Yantai Institute of Materia Medica, Yantai 264000, China; 3 Shandong Laboratory of Yantai Drug Discovery, Bohai Rim Advanced Research Institute for Drug Discovery, Yantai 264117, China

**Keywords:** drug delivery, PLGA, synthesis, biodegradable, applications

## Abstract

In recent years, biodegradable polymers have gained the attention of many researchers for their promising applications, especially in drug delivery, due to their good biocompatibility and designable degradation time. Poly (lactic-co-glycolic acid) (PLGA) is a biodegradable functional polymer made from the polymerization of lactic acid (LA) and glycolic acid (GA) and is widely used in pharmaceuticals and medical engineering materials because of its biocompatibility, non-toxicity, and good plasticity. The aim of this review is to illustrate the progress of research on PLGA in biomedical applications, as well as its shortcomings, to provide some assistance for its future research development.

## 1. Introduction

To enhance the therapeutic impact of medications and lessen potentially dangerous side effects, researchers are looking for more efficient means of drug delivery. Some of the most attractive kinds of materials are polymeric materials, which can be structurally modified. However, the focus of research has gradually shifted to absorbable and biodegradable polymeric materials due to the problems associated with the solubility, degradability, and biotoxicity of polymeric materials [1]. A vast variety of synthetic and natural biodegradable polymeric materials are available. Chitosan, sodium alginate, gelatin, albumin, and starch are typical natural biodegradable polymers [2]. Common synthetic biodegradable polymers include polylactic acid (PLA), PLGA, and polycaprolactone (PCL) [3,4,5], which are widely used in tissue engineering, therapy, diagnostics, human imaging, etc [6].

In recent years, PLGA has been extensively studied as a common biodegradable polymer that is usually broken down in the body into LA and GA, which is eventually metabolized by the body into carbon dioxide and water [7,8]. Because of its outstanding biocompatibility, biodegradability, and good mechanical properties, PLGA has received approval from the US Food and Drug Administration (FDA) as well as the European Medicines Quality Agency (EMA) as a superior drug carrier [9,10]. There are currently numerous pharmaceutical formulations based on them on the market or in clinical trials, such as microspheres, implants, gels, and others [11,12].

## 2. Methodology

The main purpose of our research was to study the application of PLGA in the biomedical field and to summarize the advantages and existing problems in its application to provide some help for its future research development. We used Web of Science, PubMed, CAS SciFinder, and other databases to search for articles written in English between 1995 and 2022 using the following keywords: Drug delivery, PLGA, Synthesis, Biodegradable, and Applications. The retrieved studies were analyzed and screened in detail, and selected references cited in the full-text downloaded studies were reviewed for other relevant studies. We selected articles that made significant research advances in the field of biomedicine in the last five years, excluding articles that applied some of these advances to other non-medical fields and those that have been in publication longer.

This paper first introduces the physicochemical properties, synthesis, and degradation characteristics of PLGA and then covers the applications of PLGA-based composites in the biomedical field in the past five years. Finally, we discuss the potential future development of PLGA and the problems that exist. In the meantime, we hope that our review will provide useful references for future research.

## 3. The Synthesis of PLGA

The biodegradable aliphatic amorphous polymer called PLGA is composed of LA that has been randomly polymerized with GA [13]. There are two classical methods for the synthesis of PLGA: ring-opening polymerization addition and direct polycondensation reactions [14,15]. The use of this approach is considerably constrained due to the necessity for metal catalysts and the elimination of leftover monomers in the manufacture of high-molecular-weight PLGAs utilizing ring-opening polymerization [16]. Although the direct polycondensation procedure is less expensive, its use is somewhat constrained due to the lesser molecular weight of PLGA that it can synthesize [14].

**Figure 1:**
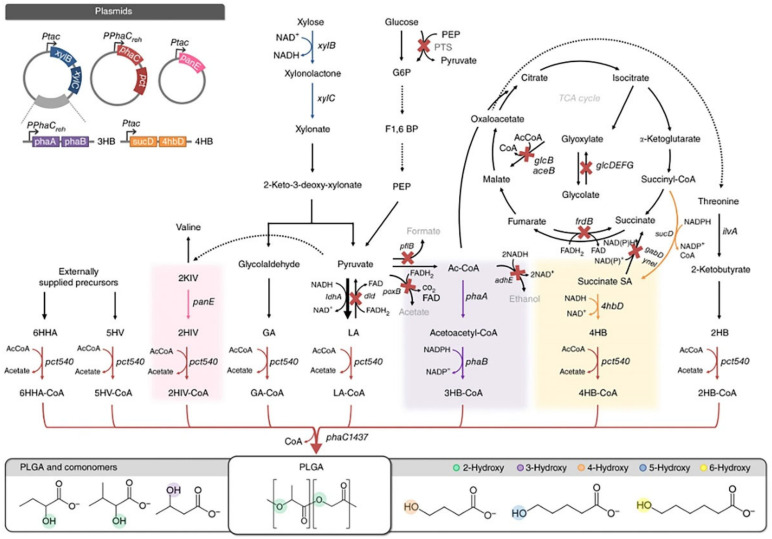
The synthesis of PLGA in Escherichia coli. Black arrows indicate the native pathways in *E. coli*; red arrows indicate heterologous pathways for polymerization of various monomers; pink, purple and orange arrows and shading indicate biosynthetic pathways for the generation of 2-hydroxyisovalerate CoA (2HIV-CoA), 3-hydroxybutyrate CoA (3HB-CoA) and 4-hydroxybutyrate CoA (4HB-CoA), respectively; blue arrows indicate the Dahms pathway. X-marks indicate inactivated metabolic pathways; bold arrows indicate metabolic pathways strengthened by replacement of native promoter. Dotted arrows indicate pathways that have been simplified from more than one conversion step. G6P, glucose 6-phosphate; F1,6BP, fructose-1,6-bisphosphate; PEP, phosphoenolpyruvate; Succinate SA, succinate semialdehyde; Ac-CoA, acetyl-CoA; LA, D-lactate; GA, glycolate; 2KIV, 2-ketoisovalerate [17]. Copyright 2016, Springer Nature.

It is crucial that materials used in biomedicine be both biosafe and biocompatible. Recently, scientists have investigated a fresh metabolic engineering approach for the synthesis of PLGA, as shown in Figure 1. In one study, Choi et al. [17] discovered a new method for the one-step generation of PLGA from carbohydrates in *Escherichia coli* (*E. coli*). The cells were made to utilize both glucose and xylose to produce D-LA and GA, and E. coli was designed to synthesize ethanolate from xylose. D-LA and GA were then transformed into their coenzyme A (CoA) intermediates in vivo by propionyl-coenzyme A transferase. Finally, these CoA intermediates were copolymerized into PLGA by polyhydroxyalkanoate synthase. This approach significantly lowered the cost of PLGA manufacture by enabling the metabolically modified strain to synthesize the numerous monomers needed for PLGA production in vivo via several metabolic pathways as compared with conventional synthesis techniques. Moreover, using microbial production methods makes it very simple to bind to other copolymer monomers, enabling the synthesis of many D-lactone copolymer types [17]. This heterologous biosynthetic pathway for the production of PLGA is a promising strategy.

## 4. The Physicochemical Properties of PLGA

Two monomers, LA and GA, are polymerized to create the biocompatible and biodegradable polymer known as PLGA [13]. Different relative molecular weight and monomer composition ratios primarily affect the physicochemical characteristics of PLGA [18]. Typically, the higher the level of GA, the faster the degradation of PLGA. Therefore, in the realm of biomedical research, greater focus has been placed on choosing the right LA to GA ratio in order to control the degradation kinetics of in vivo delivery systems [19]. In biomedical applications, PLGA typically has a molecular weight of 5–40 kDa [9]. In addition, the viscosity of PLGA and its molecular weight are closely related [19]. The glass transition temperature (Tg) of PLGA copolymers, which are intrinsically amorphous, ranges from 45–55 °C [19]. Since PLGA is soluble in numerous organic solvents, including dichloromethane, tetrahydrofuran, ethyl acetate, trichloromethane, acetone, and benzyl alcohol [13], various PLGA-based biomaterials can be constructed using various methods and procedures.

The main elements determining the drug-release system are the crystallinity, Tg, characteristic viscosity, and relative molecular mass of PLGA. The crystallinity of PLGA impacts its mechanical strength, swelling, and biodegradation rate. The crystallinity of the polymer itself depends on the monomer units formed during the copolymerization process between GA and LA [20]. Modifying the composition of a polymer can result in a variety of property changes that may ultimately have an impact on drug release and polymer degradation, among other issues [19]. It has been reported that a decrease in LA content leads to a decrease in Tg, and a decrease in Tg causes the polymer matrix to plasticize; these modifications cause the mechanical characteristics of PLGA to decline [21]. Consequently, Tg is directly correlated with both the relative molecular mass and composition of the copolymer [19,20]. Additionally, the contact surface area and the release of highly hydrophilic medicines are closely correlated with the degradation of PLGA. Furthermore, chemical interactions between the polymer matrix and the medication may cause higher rates of degradation [22]. To adjust the system degradation and drug release mechanisms, all parameters that have an impact on the complete polymer system that is used must be taken into consideration.

## 5. The Degradation Properties of PLGA

**Figure 2:**
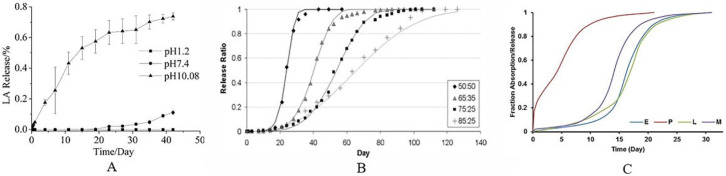
(**A**), Comparison of PLGA degradation rates under different pH conditions [30]. Copyright 2008, Wiley. (**B**), Comparison of PLGA degradation rates for different monomer ratios [31]. Copyright 2010, Springer Nature. (**C**), Comparison of the degradation rates of PLGAs with different intrinsic viscosities; the magnitude of the intrinsic viscosities of the four PLGAs is P < L < E < M [32]. Copyright 2022, Elsevier.

The primary processes of PLGA degradation are hydrolytic ester bond degradation and autocatalytic degradation [13,23]. Table 1 lists the pertinent factors influencing PLGA degradation as well as their occurring mechanisms. Some of the relevant factors affecting the degradation characteristics of PLGA are listed in Figure 2. In an aquatic environment, PLGA degrades via hydrolysis as follows: Initially, water permeating into the amorphous regions of the polymer matrix lowers its Tg, breaking the van der Waals forces and hydrogen bonds. In turn, the molecular weight of the polymers is reduced, which raises their hydrophilicity and speeds up the breakdown of the polymers into water-soluble fragments. This process is caused by the continuous breaking of covalent bonds between the polymers. Finally, these fragments are hydrolyzed into LA and GA, which are then broken down by normal metabolic pathways into energy, CO_2_, and water [13,24]. Since the methyl group on the side chain and poly (lactic acid) are more hydrophobic than poly (glycolic acid), by adjusting the ratio of LA to GA monomer, which degrade the fastest when the ratio is 50:50, the degradation rate may be variably regulated [25,26]. In addition, PLGA can undergo autocatalytic degradation. The slightly acidic local microenvironment of the organism can be brought on by the acidic degradation products produced during its degradation, which could speed up degradation [27]. However, the acidic environment caused by PLGA degradation may lead to local inflammatory reactions in the body [28], and it might have a negative impact on how long encapsulated pharmaceuticals stay stable; particularly, how long therapeutic proteins stay stable [29]. In order to improve the stability of medications and provide better therapeutic effects, the pH at local tissues can be controlled by adding a less water-soluble alkaline material.

## 6. The Applications of PLGA to Biomedical Research

In recent years, the development of biomolecular therapeutics has raised the demand for designing efficient and highly specific drug delivery strategies to ensure optimal bioavailability of encapsulated therapeutics for optimal therapeutic efficacy. Due to its diversity in formulation, functionalization, biodegradability, biosafety, and biocompatibility, PLGA is among the most developed polymers for modern medical applications [33]. Currently, PLGA-based pharmaceutical formulations have been extensively researched and developed. This section provides a brief review of the current status of their research in biomedicine.

### 6.1. In-Tumor Diseases

Given their millions of fresh instances each year, tumors are one of the deadliest and most serious diseases in the world. Examples of conventional treatments include surgery, radiation therapy, and chemotherapy. The employment of standard therapy techniques, however, might have major negative effects on patients. Additionally, conventional therapy failure can increase the risk of tumor metastasis and recurrence [34]. The utilization of nanoparticles has recently offered a solid foundation for effective drug delivery and monitoring systems due to our growing understanding of the interactions between microparticles (MPs), nanoparticles (NPs), biological tissues, and the tumor microenvironment [35].

**Figure 3:**
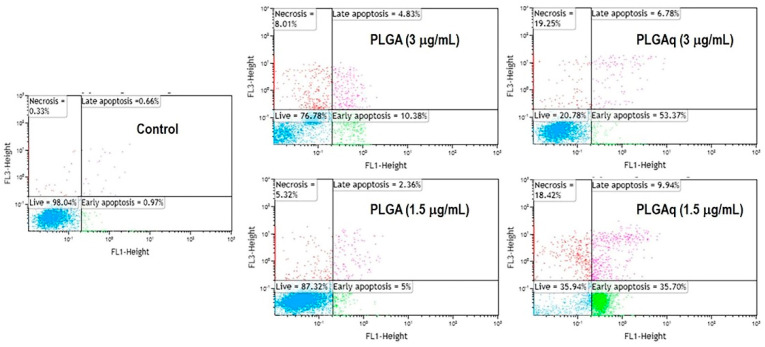
Effect of PLGAq on the induction of apoptosis in MCF-7 cell line detected by Annexin V-FITC and PI double-staining method. At a concentration of 1.5 μg, the early and late apoptosis rates of PLGA microspheres were 5% and 2.36% respectively; at a concentration of 3.0 μg, the early and late apoptosis rates of PLGA microspheres were 10.38% and 4.83% respectively. At a PLGAq concentration of 1.5 μg, 35.70% and 9.94% apoptosis (early and late) was observed. When the concentration was increased to 3.0 μg, the apoptosis rate (early stage) was 53.37% and 6.78% in the late stage. A significant increase in the number of apoptotic cells was observed with increasing concentrations of PLGAq after administration. [45]. Copyright 2019, Elsevier.

In the treatment of oncological diseases, drugs are more effectively administered and less harmful when they are specifically directed to cancer cells using nanocarriers. To control medication distribution and drastically reduce the incidence of cardiomyopathy during application, Park et al. [36] applied polyethylene glycolic PLGA NPs to encapsulate adriamycin. Glioblastoma multiforme (GBM) is one of the most common and aggressive malignancies [37,38,39]. Drug molecules typically encounter the blood–brain barrier during drug delivery, and repeated drug administration can result in tumor resistance and ineffective tumor clearance [40]. Verteporfin (VP) inhibits the proliferation of GBM cells while improving the radiosensitive phenotype of these cells. In order to generate biodegradable MPs for the treatment of GBM, Shah et al. [41] encapsulated VPs with PLGA matrix. They implanted MPs into mice with established tumor models, and tumor growth was significantly inhibited in mice in the VP–PLGA group compared to the control group. On the other hand, the brain is a very fragile and unstable organ that tolerates only minimal trauma; as PLGA MPs are not only capable of loading sufficient doses of drugs but are also long-lasting, slow-release, and biodegradable, they are very useful in the treatment of brain diseases. The life and health of women are significantly threatened by breast cancer, one of the most prevalent malignant tumors in women [42,43,44]. Quercetin–PLGA MPs (PLGAq) were loaded with PLGA MPs by Karthick et al. [45], and the results of cytotoxicity assays revealed that they were not toxic to normal cells (THP-1 cell line), while in mammary cells (MCF-7 cell line), PLGAq demonstrated effective antitumor activity at concentrations of 1.5–3 g/mL. At higher concentrations, PLGAq significantly inhibited the growth of MCF-7 cell lines in flow cytometry experiments (Figure 3). These studies have shown the potential of PLGA-based drug formulations for the treatment of tumor-like disorders by demonstrating improved efficacy with more accurate drug delivery and reducing undesirable effects brought on by elevated toxicity of chemotherapeutic medicines.

### 6.2. In Neurodegenerative Diseases

**Figure 4:**
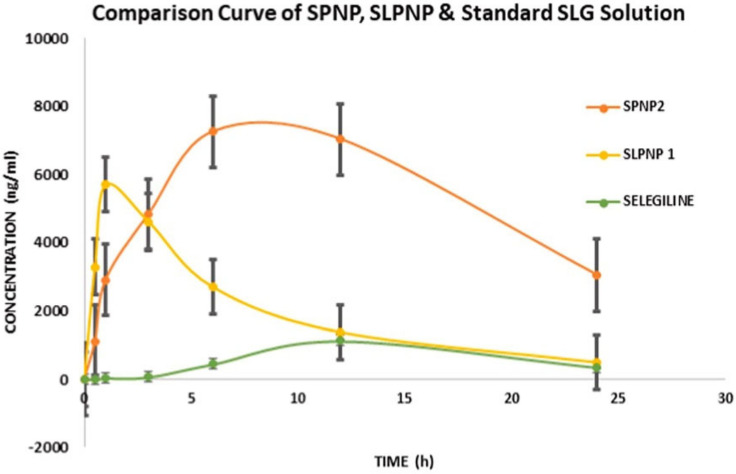
Concentrations of selegiline in rat brain tissue after administration of different dosage forms, SPNP is a selegiline PLGA nanoparticle, SLPNP is a Phospholipon^®^ 90 G modified selegiline PLGA nanoparticle [51]. Copyright 2022, Elsevier.

Neurodegenerative diseases are diseases of irreversible and progressive loss of nerve cells. They include Alzheimer’s disease (AD) [46] and Parkinson’s disease (PD) [47], can lead to severe disability, and have a huge social and economic impact. The selectivity of the blood–brain barrier, which significantly lowers the therapeutic efficacy of medicine, is the main barrier to effective drug administration. Due to their controlled and sustained release effects, low cytotoxicity, superior biocompatibility, and targeted delivery, PLGA NPs are often utilized in CNS-targeted drug delivery. Szymusiak et al. [48] encapsulated the highly hydrophobic curcumin in PLGA NPs, which improved oral absorption and decreased the drug dose necessary to achieve comparable plasma and nervous system tissue concentrations in mice after oral administration by approximately twofold compared to unencapsulated curcumin. In addition, a promising strategy for the treatment of neurodegenerative conditions like AD was discovered by Tiwari et al. [49], who discovered that PLGA-encapsulated curcumin improved brain self-healing mechanisms and restored abnormalities in hippocampus neurogenesis and learning memory loss in a rat model of AD. Barcia et al. [50] developed a novel drug delivery system for the treatment of PD that encapsulates ropinirole in PLGA NPs, and behavioral testing experiments in rats showed that the system was able to restore PD symptoms in neurodegeneration. Selegiline PLGA nanoparticles (SPNPs) were prepared by a single emulsion dissolution evaporation method by Raman et al. [51]. In vitro studies showed a drug penetration rate of 77.56% for SPNPs, compared to 65% for pure selegiline. In vivo experiments have shown that SPNPs have stable controlled-release properties with a half-life of approximately 13.5 h and higher drug concentrations in rat brain tissue compared to pure selegiline administration (Figure 4); therefore, SPNPs are promising carriers for the intranasal delivery of selegiline and can effectively improve its brain bioavailability. By loading drugs into PLGA NPs, it is possible to overcome the limitations of traditional forms of drug delivery and significantly improve the ability of drugs to penetrate the blood–brain barrier, allowing them to be targeted to localized lesions, significantly improving their therapeutic efficacy and making them a very promising strategy for the treatment and prevention of neurodegenerative diseases.

### 6.3. In Pulmonary Diseases

**Figure 5:**
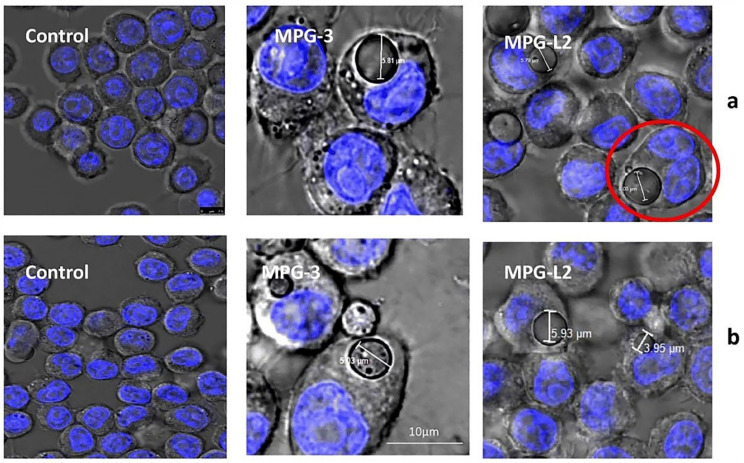
Phagocytosis of MPs by macrophages at 24 h (**a**) and 48 h (**b**). The red circles indicate that MPs do not influence macrophages to replicate while inside the cell [57]. Copyright 2020, Springer Nature.

As a result of modern formulations like MPs, which may carry medications directly to the lungs to treat pulmonary disorders, drug therapy is now more effective, systemic toxicity is decreased, and patient compliance is increased [52]. The third-most common cause of mortality worldwide is a dangerous lung condition called chronic obstructive pulmonary disease (COPD) [53]. The current treatment of COPD mainly uses anti-inflammatory, antioxidant, and corticosteroid drugs, but the process of application requires higher doses and serious adverse effects [54]. Small molecule heat shock protein α,β-crystallin (HSPB5) is an extracellular protein with anti-inflammatory properties in a majority of inflammatory models [55]. However, the body possesses natural anti-HSPB5 antibodies to block its binding to the receptor, and Johannes et al. [56] encapsulated HSPB5 in porous PLGA MPs, which effectively protected HSPB5 from neutralization, while PLGA MPs stimulated the phagocytosis of HSPB by macrophages. In contrast to a 30-fold dose of free HSPB5 that remained ineffective, alveolar macrophages in COPD model mice selectively took up PLGA MPs loaded with HSPB5 when delivered via the lung, significantly inhibiting lung invasion by inflammatory cells. Similar to the previous investigation, Marcianes et al. [57] used Labrafil to surface-modify PLGA MPs and targeted gatifloxacin to macrophages for the treatment of tuberculosis. They reported that in the study of the phagocytic behavior of macrophages, surface-modified MPs could be phagocytosed within a short period, and after co-incubation for 48 h, macrophages were still alive, and MPs were visible inside the cells (Figure 5). While also enhancing their targeting from macrophages, these MPs overcome the disadvantage of excessively rapid drug release and extend the therapeutic window of the drug. Although the pharmacokinetic features of PLGA MPs in the respiratory system still need to be further investigated, they exhibit enormous promise as an inhaled delivery mechanism for the treatment of respiratory illnesses.

### 6.4. In Bone Tissue Engineering

**Figure 6:**
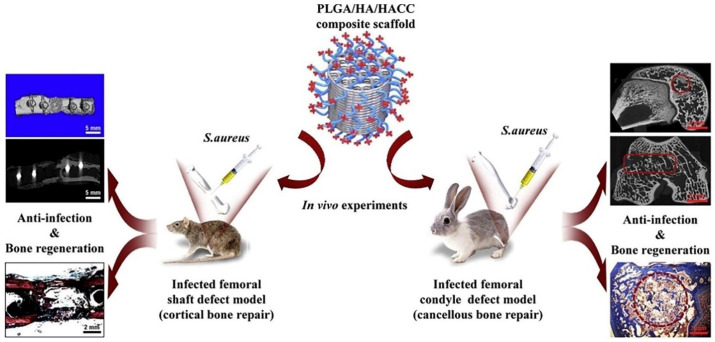
HACC–PLGA/HA composite scaffold in cortical bone defects in rats and hairy bone defects in rabbits [68]. Copyright 2018, Elsevier.

Clinical management of bone abnormalities and the illnesses they are associated with continue to be quite challenging. Bone grafting is usually the mainstay of clinical treatment for bone defects, which can be regenerated to some extent by using different grafts. Autologous bone grafts have osteoconductive and osteoinductive capabilities [58]; however, their availability and donor site morbidity continue to constrain their utilization [59]. Allografts run the risk of transmitting infectious diseases and triggering an immunological response [60]. High-biocompatibility artificial bone substitutes are currently receiving more attention. Because of its superior biocompatibility, tunable biodegradability, and mechanical characteristics, PLGA has been employed extensively in bone tissue engineering [61,62]. Additionally, it can promote the electrical conductivity of bone scaffold materials, promote osteoblast adherence and value addition, and stimulate mesenchymal stem cell differentiation into osteoblasts [63]. In a recent study, Esrafilzadeh et al. [64] created multifunctional graphene–PLGA fibers with exceptional mechanical properties, cellular affinity, and electrical conductivity. These fibers also had a significantly higher Young’s modulus and tensile strength than previously reported nano-carbon–PLGA fibers. Due to its advantages in accurate manufacturing, rapid prototyping, and customizable structures, over the past two decades, 3D printing has become increasingly applied in various tissue engineering and regenerative medicine contexts [65,66,67]. Yang et al. [68] created PLGA/hydroxyapatite (HA) 3D-printed scaffolds with quaternized chitosan (HACC)-grafting for the treatment of significant bone deficiencies following trauma or tumor resection (Figure 6). In their experiments, PLGA/HA composite scaffolds with HACC grafts shown noticeably enhanced anti-infection and bone regeneration efficacy in both rat cortical bone defects and rabbit hairy bone defects. For spinal fusion treatment, Lin et al. [69] generated a PLGA/tricalcium phosphate (β-TCP) scaffold that was loaded with salvianolic acid B. In animal experiments, salvianolic acid B has been found to have a dose-dependent effect on the rate of mineral adsorption and angiogenesis. The PLGA/β-TCP composite scaffold loaded with salvianolic acid B promoted osteogenesis and angiogenesis to enhance bone fusion. There is still more to be accomplished before PLGA-based skeletal materials may be utilized in clinical situations, despite recent studies demonstrating that they have high biocompatibility, angiogenesis, and osteogenesis.

### 6.5. In Ocular Disease

**Figure 7:**
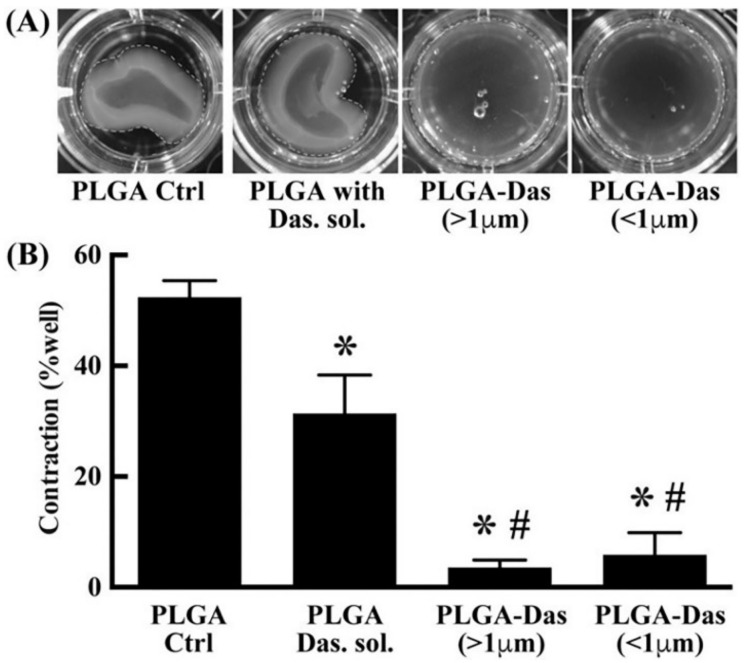
Collagen matrix contraction assay. (**A**) Collagen matrix contraction images. (**B**) Quantitative data of collagen contraction. *n* = 5; * *p* < 0.05: significant difference from the control group; ^#^ *p* < 0.05: significantly different from the PLG–Adasatinib group [76]. Copyright 2019, Elsevier.

Static barriers like the corneal layer, retina, blood–aqueous barrier, and blood–retinal barrier, as well as dynamic barriers like tear dilution and lymphatic clearance, are frequently found in the administration of ocular drugs [70,71,72]. Due to the existence of these barriers, it might be challenging to maintain an adequate drug concentration due to unequal therapeutic drug distribution, and major side effects such as cataracts and retinal detachment can occur during treatment [73]. As a result, a continuous-release drug delivery system (DDS) that is secure and safe must be created in order to treat ocular illnesses. A glucocorticoid called triamcinolone acetonide is frequently used to treat a variety of inflammatory ocular illnesses, because it has the potential to effectively restrict the generation of vascular endothelial growth factor and cytokine production and reduce vascular permeability [74]. Triamcinolone acetonide was encapsulated in chitosan-coated PLGA NPs by Dandamudi et al. [75], and they reported that a plateau could be reached 27 h after administration. The medication was then continuously delivered in a low-burst form to improve bioavailability. In a different study, Chauhan et al. [76] investigated the development of PLGA MPs that were loaded with dasatinib for the treatment of value-added retinopathy. Microspheres >1.0 μm in diameter showed sustained drug release for 55 days. In addition, in an in vitro scar contraction experiment, dasatinib-encapsulated PLGA MPs dramatically decreased collagen matrix contraction (Figure 7), and the therapeutic effect of dasatinib-loaded PLGA microspheres was found to be superior to that of dasatinib alone in experiments simulating the ocular environment. Additionally, it is critical to address the limited bioavailability of ocular drug delivery. Vasconcelos et al. [77] coupled PLGA–polyethylene-glycol (PEG) NPs with human immunodeficiency virus trans-activator and peptide for ocular delivery (POD) to obtain PLGA–PEG–POD NPs with smaller particle size, higher encapsulation rate, and better-sustained release performance. Meanwhile, the positive charge on the surface of NPs combined with the positively charged peptide-promoted penetration into corneal epithelial cells, which greatly improved the bioavailability of ocular drugs. In summary, PLGA-based DDSs show excellent promise for ocular drug delivery because of their biodegradable and biocompatible properties as well as their capacity for effective and secure drug administration.

### 6.6. In Diagnostic

**Figure 8:**
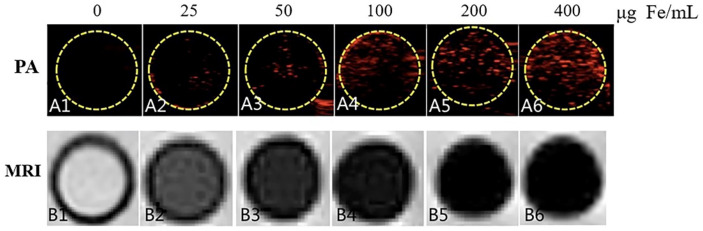
Dual-modal PA and MRI imaging ability of magnetic PLGA-IO MPs. Qualitative observations show an enhanced PA signal with increasing iron content in the particles and a low signal on MRI [84]. Copyright 2018, Public Library of Science.

Currently, magnetic resonance imaging (MRI), computed tomography (CT), and ultrasound imaging are the principal diagnostic techniques frequently utilized in clinical practice [78]. In recent years, the use of contrast agents in illness detection and therapy has steadily increased, because they can achieve high spatial resolution and high sensitivity for disease imaging [79,80]. However, the drawbacks of contrast agents, such as poor stability, a rapid in vivo clearance rate, and a lack of targeting, restrict their applications [81], and encapsulating contrast agents in a PLGA-based DDS can enhance their targeting capability and biocompatibility [79,82]. Usually, combining PLGA with inorganic nanoparticles (INP) with magnetic properties produces an enhancement of MRI signals. pH-sensitive PLGA nanoparticles were prepared to encapsulate manganese oxide (MnO) by Bennewitz et al. [83] Polymer particles under physiological conditions do not release Mn^2+^ and have almost no contrast in MRI. The MRI properties can vary up to 35-fold with a more prominent contrast in acidic environments; however, when the polymer particles are worn down and MnO dissolves, Mn^2+^ is released. Recently, Lu et al. [84] developed contrast-agent–PLGA–iron-oxide particles (PLGA–IO MPs) for photoacoustic tomography (PA) with MRI dual-modality tracking of tendon stem cells (TSCs). As observed by fluorescence staining and transmission electron microscopy, PLGA–IO MPs had good PA/MRI tracer TSCs at relatively low iron concentrations, and the signal of PA with MRI was higher as the iron concentration increased (Figure 8). Although it is still in the preclinical research stage, the application of PLGA in conjunction with INP for the diagnosis of diseases is a very promising field and is now utilized to help study major diseases including cancer and neurodegenerative diseases. As a result, it is critical to keep investigating and developing effective PLGA–INP for therapeutic use.

### 6.7. In Immunomodulation

**Figure 9:**
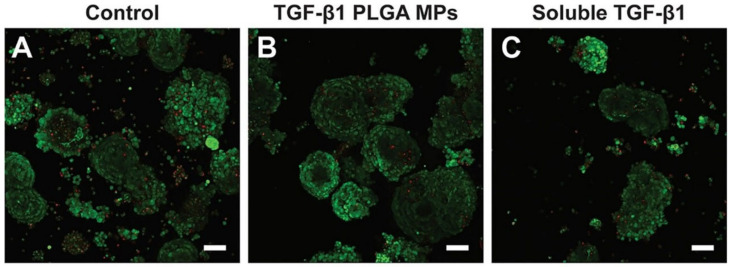
The biocompatibility study of TGF-β1/PLGA MPs with rat islet cells, (**A**) control islets, (**B**) islets incubated with TGF-β1 PLGA microparticles, (**C**) islets incubated with soluble TGF-β1, Red = dead cells, Green = viable cells [90]. Copyright 2021, Frontiers.

In the past decades, organ tissues, including the kidney, liver, and heart, have been successfully transplanted [85], but there can be severe organ rejection in organ transplantation. The immunosuppressive drugs currently used have the disadvantages of low specificity and causing serious adverse effects [86]. However, researchers have shown that micro-nanoparticles, for instance, can target, maintain, and control the release of medications to improve therapeutic effect, somewhat addressing the shortcomings of immunosuppressive drugs [87]. Allogeneic islet transplantation is a highly promising therapeutic approach for the management of type 1 diabetes mellitus [88]. The drawbacks of systemic immunosuppression, host-mediated immunological rejection, and shortage of donor cells, however, severely restrict application [89]. Li et al. [90] used PLGA MPs to encapsulate transforming growth factor-β1 (TGF-β1), thereby achieving its localization and controlled release. They co-transplanted TGF-β1-loaded PLGA MPs with mouse islets in a diabetic mouse model with favorable biocompatibility and detectable insulin expression in the peripheral sites of the long-term functioning grafts (Figure 9). The results of studies on the extensive impacts of TGF-β1/PLGA MPs on the host immune system suggest that this immune regulation is localized. In conclusion, the use of a PLGA-based DDS, which is suitable for the local graft microenvironment to provide drugs, can effectively improve the targeting performance of drugs, reduce systemic toxicity after drug administration, and reduce the occurrence of graft rejection.

### 6.8. In Inflammatory Diseases

**Figure 10:**
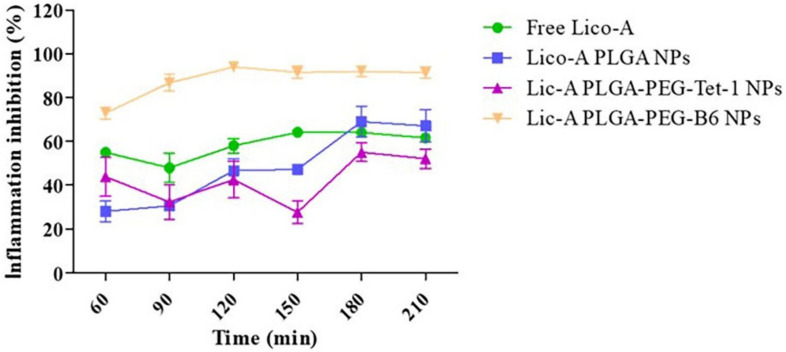
Inhibitory ability of different doses of licochalcone-A on ocular inflammation [96]. Copyright 2022, MDPI.

The inflammatory response is a defense mechanism that the tissues of the body activate when they sustain harm, but it may also obliterate tissue structure and cause lesions or necrosis [91]. To lessen the danger of severe side effects related to the long-term systemic administration of medications for the treatment of inflammatory disorders, topical administration may be a viable option. The development of new anti-arthritic medications should be a top priority, because arthritis is one of the illnesses that causes the most suffering in humans. Naringin (NAR) is a flavonoid that is present in grapefruit and herbs and has pharmacological properties that include anti-inflammatory, antioxidant, and hypolipidemic [92,93]. In recent work, Mohanty et al. [94] performed in vivo anti-arthritis investigations in a mouse model after first encapsulating NARs with PLGA NPs. The experimental findings demonstrated that, as compared to blank NPs and free medication at a dosage of 20 mg/kg, NAR–PLGA NPs considerably increased anti-inflammatory capability, decreased rheumatoid factor (RF) and C-reactive protein (CRP) levels, and efficiently increased the bioavailability of NAR. One of the most prevalent inflammatory disorders that can harm different areas of the eye and surrounding tissues to differing degrees is inflammation of the eye [95]. The natural anti-inflammatory licochalcone-A has limited water solubility, which frequently prevents its application. It was loaded into polyethylene glycolyzed PLGA nanoparticles and modified with cell-penetrating peptides (B6 and Tet-1) by Galindo et al. [96] to alleviate eye inflammation. The experimental results showed that PLGA NPs, when surface-functionalized using cell-penetrating peptide B6, had a stronger inflammatory inhibitory effect compared to other forms of drug delivery, effectively avoiding rapid clearance of the drug by the organism and improving its targeting (Figure 10). The bioavailability of anti-inflammatory medications can be significantly increased by combining PLGA with drugs, providing important benefits during the treatment of inflammation and offering promising potential for topical administration. However, the acidic environment produced in the degradation of PLGA may have an impact on the treatment of inflammation, which is a side effect that needs to be further addressed.

### 6.9. In Cardiovascular Diseases

**Figure 11:**
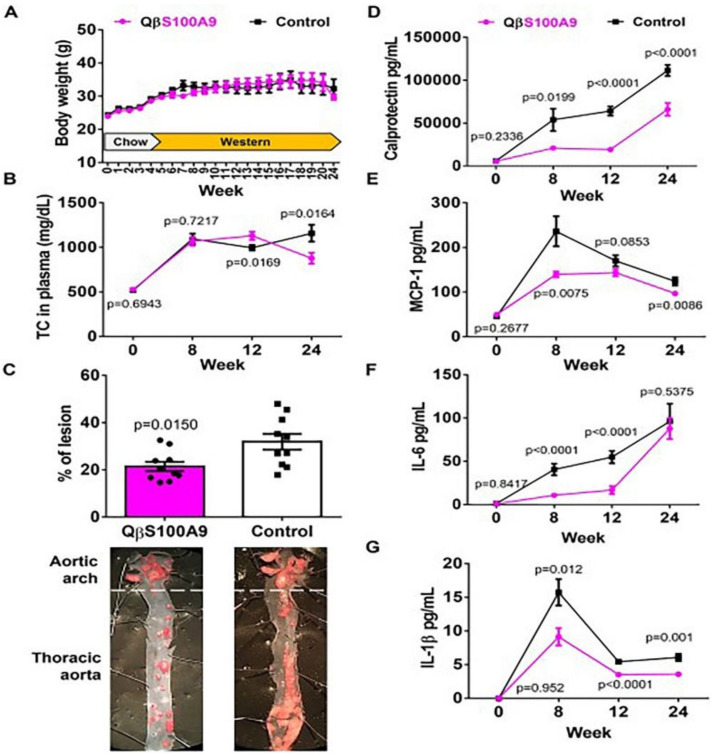
Effect of QβS100A9 vaccine implantation on atherosclerosis in mice on a high-fat diet, (**A**) Body weight (grams), (**B**) The concentration of total cholesterol in plasma., (**C**) The presence of atherosclerotic plaques shown as the percentage of lesion determined by oil red O staining., (**D**) Plasma concentrations of calprotectin, (**E**) Plasma concentrations of MCP-1, (**F**) Plasma concentrations of IL-6, (**G**) Plasma concentrations of IL-1β. Data are means ± SEM (*n* = 10), unpaired two-tailed *t*-test, 95% confidence value, *p* < 0.05 was considered the threshold for statistical significance versus control group [99]. Copyright 2022, Wiley.

More than 60 million individuals in Europe are at risk of mortality from cardiovascular disease (CVD), which continues to be one of the main causes of mortality worldwide [97]. The main pathogenic processes of atherosclerosis (AS), which contribute significantly to the majority of CVD cases, are interactions between immune cells, endothelial cells, and lipids [98]. Statin usage to decrease plasma cholesterol levels is the usual therapy for CVD; however, this treatment requires lifetime adherence. Ortega-Rivera et al. [99] developed a vaccine that induces targeting of the S100A9 protein, offering an alternative to the repeated use of statins as a therapeutic measure. The S100A9 polypeptide epitope-displaying Qβ-phage virus-like particles (VLPs) were used to construct the vaccine as a PLGA–VPL implant. In an animal model of cerebrovascular illness, the implant considerably decreased the levels of serum calcineurin, Interleukin-1β (IL-1β), Interleukin-6 (IL-6), and monocyte chemoattractant protein-1 (MCP-1), offering long-lasting relief from atherosclerosis (Figure 11). On the other hand, blood clots can also cause a range of CVD [100]. Streptokinase (SK) and tissue-type fibrinogen activator (t-PA) are two of the most effective thrombolytic medicines for treating thrombosis and enhancing patient survival. Hasanpour et al. [101] wrapped SK in mPEG–PLGA NPs to prolong its in vivo action. Results from in vivo studies showed good biocompatibility with no histopathological changes in the liver or kidneys even after 21 days of administration. In a recent study, Zamanlu et al. [102] prepared PEG–PLGA NPs loaded with t-PA using a single emulsion solvent diffusion/evaporation technique to prolong the circulation time and thrombolytic activity of t-PA and enhance its targeting ability. Compared to normal t-PA solutions, t-PA–PEG–PLGA NPs exhibit higher thrombolytic activity, up to 2–6 times that of free t-PA. In conclusion, the use of PLGA-based DDSs has tremendous potential for the treatment of CVD, since they can increase patient compliance, target performance, and duration of effect of medicines.

### 6.10. In Infection

**Figure 12:**
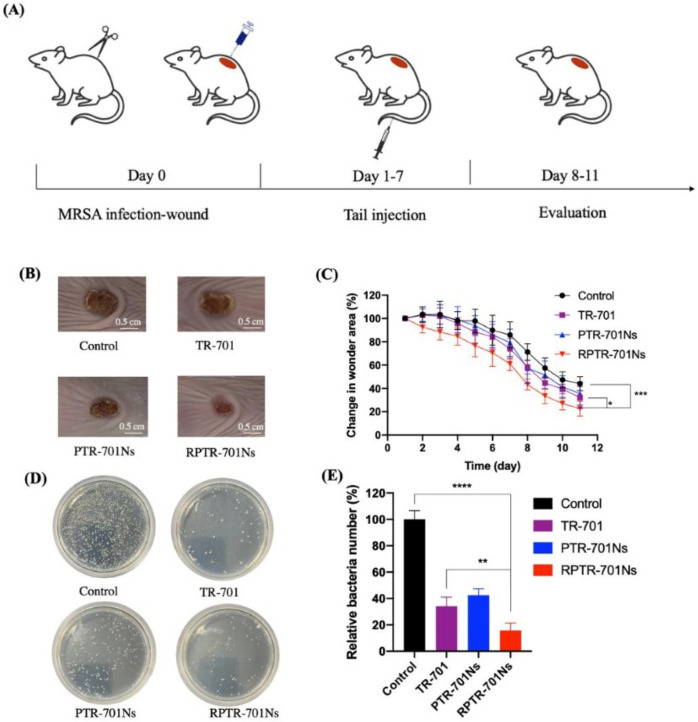
In vivo antibacterial effect of RPTR-701 NPs on MRSA-infected mice, (**A**) Schematic diagram of MRSA-infected model establishment and treatment plan, (**B**) Wound skin after treated with PBS, TR-701, PTR-701Ns and RPTR-701Ns, (**C**) Relative change in the wound area, (**D**) LB culture pates of different groups of skin, (**E**) Quantitative analysis of bacteria colony, Data were presented as mean * *p* < 0.1, ** *p* < 0.01, *** *p* < 0.001, **** *p* < 0.0001 [106]. Copyright 2021, MDPI.

Antibiotic abuse leads to multidrug resistance (MDR) in bacterial infections, which is already a significant, global problem in clinical treatment [103]. Methicillin-resistant Staphylococcus aureus (MRSA) is a group of bacteria that are highly virulent and can cause serious diseases such as pneumonia, purulent skin, and soft tissue infections [104]. Tedizolid phosphate (TR-701) is a novel antibiotic for the treatment of acute bacterial skin and tissue infections caused by MRSA [105]. Wu et al. [106] explored the encapsulation of PLGA NPs loaded with TR-701 (RPTR-701 NPs) with red blood cell membranes (RBCM). Their RPTR-701 NPs had a 192.50 ± 5.85 nm bilayer nuclear structure, which significantly reduced phagocytosis of RAW 264.7 cells due to the presence of RBCM. On the other hand, this approach helped to reduce damage to red blood cells via MRSA exotoxin and promoted wound healing due to a strong exotoxin-neutralizing ability (Figure 12). Polymeric film-forming systems (FFSs) are currently a subject of considerable interest for their ability to control the release of drugs at a target site on the skin, thereby improving therapeutic efficacy [107]. Snejdrova et al. [108] prepared FFSs loaded with terbinafine hydrochloride (Ter-HCl) for the treatment of superficial fungal infections using PLGA and the plasticisers ethyl pyruvate and methyl salicylate.

## 7. Prospectives and Research Gaps

The approval of PLGA by the FDA and EMA for a variety of medical applications is one of its key benefits, and gives PLGA-based materials significant advantages for clinical trials and applications.

Because of its superior mechanical properties, strong biodegradability, and biocompatibility, PLGA is recognized as a suitable DDS for the controlled-release drug delivery of medicines, peptides, proteins, and other substances. Generally speaking, the monomer composition, molecular weight, Tg, and characteristic viscosity of PLGA all have a significant impact on its characteristics. These factors also have an impact on the drug-loading capacity, when PLGA is used as a drug-delivery matrix, and drug-release characteristics. As a result, when selecting various PLGA types, researchers should take their physicochemical characteristics into account. Another important factor to consider is the physicochemical profile of the drugs themselves. The structural and functional characteristics of PLGA matrices can be used in the future to build PLGA polymers with a particular application or degradation kinetics.

By specifically functionalizing the polymeric MP/NP surface, it is possible to limit entry into healthy cells and increase biodistribution in the target tissues and organs. At the same time, the use of the PLGA matrix as a contrast agent carrier can significantly increase the precision of major disease diagnosis and offer a more efficient treatment for many diseases that are challenging to treat. More research is needed before practical clinical applications.

Degradable biomaterials based on PLGA have advanced significantly in tissue engineering research in recent years due to the continued advancement of biodegradable materials research and growing demand. In order to increase their applicability in clinical treatment, basic research on the interaction between the material and the organism could be conducted in the future to assess potential benefits and drawbacks. In addition, the low Tg of PLGA indicates that appropriate sterilizing methods, including autoclaving and radiation sterilization, must be chosen for tissue-engineering applications.

On the other hand, biomimetic DDSs using PLGA as a substrate and modified with cell membranes (CM) are also becoming a popular topic of research [109]. The use of CM coatings can inhibit the sudden release of PLGA NPs to some extent, enhancing immune escape from the organism and improving its targeting.

Based on the available literature, we have found that in vivo degradation of PLGA-based DDS may cause changes in the local microenvironment of tissues, which may cause problems such as histopathology during relevant treatments, but this drawback has not been explored in an effective manner and requires further research. At the same time, there is a gap between basic research on and product development of PLGA MPs and NPs, and more research is needed to optimize and evaluate their performance. In addition, the analytical methods for the structure of PLGA molecules are not yet perfect and more research on this issue is needed as well, especially for the quality control of PLGA derivatives. Most importantly, the mechanism of interaction between PLGA and drugs is still unclear, and more theoretical and experimental studies are needed to reveal the effect of PLGA on the drug-release rate, stability, and bioavailability.

## 8. Conclusions

In this paper, we reviewed the synthesis process, physicochemical properties, bio-degradation characteristics, and factors affecting the biodegradation of PLGA. More importantly, we describe its recent applications to tumor diseases, neurodegenerative diseases, pulmonary, bone tissue engineering, ocular disease, diagnostic, immunomodulation, inflammatory disease, cardiovascular disease, infection, and other conditions. Many of the examples in this review demonstrate that by utilizing PLGA-based DDSs, it is possible to effectively improve the bioavailability of drugs, reduce adverse effects, and reduce the frequency of dosing, which may improve patient compliance with medication. However, these systems still have disadvantages, such as low drug-loading capacity, high production costs, and difficulties with large-scale production. It is due to these shortcomings that many studies are still at the basic research stage and lack extensive clinical trial data to assess their practical application. Therefore, we hope to further accelerate this research and development process to speed up the translation of the results of basic research into clinical treatment.

## Figures and Tables

**Table 1:** The factors and mechanisms affecting the degradation of PLGA.

Influence Factors	Performance	Mechanism
LA: GA ratio	The higher the LA ratio, the slower the degradation	The higher the percentage of LA, the more hydrophobic it is and the slower the degradation rate
End group	Acid-terminated degrades more quickly than ester-terminated	Highly hydrophobic PLGA with ester-capped end
Molecular weight	The higher the molecular weight, the slower the degradation	The larger the molecular weight, the longer the polymer chain and the slower the degradation
pH	Slower rate of degradation under alkaline conditions compared to acidic conditions	The H^+^ produced by degradation is neutralized with OH^−^ in the environment, making the autocatalytic effect of -COOH weaker
Temperature	The higher the temperature, the faster the degradation rate	Increased temperature promotes hydration layer formation

## Data Availability

Data sharing not applicable.
